# The Impact of Breakdown in Filiation: The Instance of Children Exiled From Reunion Island to Mainland France Between 1962 and 1984

**DOI:** 10.3389/fpsyg.2021.623653

**Published:** 2021-09-20

**Authors:** Marion Feldman, Malika Mansouri

**Affiliations:** ^1^UFR SPSE, UR CLISPYD 4430, Unité de Formation et de Recherche Sciences Psychologiques et Sciences de l’Education, Université Paris Nanterre, Nanterre, France; ^2^UR 4430 CLIPSYD, Clinique Psychanalyse Développement, A2P, Approche en Psychopathologie Psychanalytique, Nanterre, France; ^3^Institut de Psychologie, Université Paris Descartes, Boulogne-sur-Seine, France; ^4^PCPP EA4056, Institut de Psychologie, Boulogne-sur-Seine, France

**Keywords:** children, Reunion Island, trauma, collective history, group

## Abstract

The aim of this article was to show the consequences of breakdowns in filiation among people born between 1950 and 1970 on Reunion Island, who experienced particular traumas during their childhood. The research participants included 2,015 children exiled from Reunion Island to mainland France between 1962 and 1984 as part of a political project. Most of them we adopted, others were placed in foster families, foster homes, or farms. The forced exile was orchestrated by the French social services in charge of child protection (DDASS). Structured interviews were conducted for 13 people exiled when they were between 2 and 15years old. The interviews were transcribed and then analysed. The results show that these children were exposed to abuse in their filiation through a series of traumas. For them, this abuse is still active today as the French government has not yet acknowledged the suffering of these children. The participants displayed numerous psychic disorders linked to their abandonment. They are still experiencing difficulties in assuming their identity, and these difficulties are transmitted to the next generation. Analysis of the impact of these filiation breakdowns leads the present researchers to suggest a specific clinical setting, based on a focus group, in other words a group therapy aiming to generate a group narrative process.

## Introduction

Between 1962 and 1984, 2015 children aged from 2 to 17 were transferred to mainland France as part of a political project (Feldman, 2018). At that time, Michel Debré was the MP for Reunion. He wanted to instate a powerful executive, as had been the case with Algeria, where he had been strongly in favour of maintaining a French colonial presence, before Algeria gained independence that same year.

In 1960s, Reunion, a French *département* since 1946, was faced with many difficulties: a demographic explosion, a serious economic situation with a lack of qualified manpower and great inequalities in income (Ascaride et al., 2004, p.49). At the same time, rural zones in mainland France were experiencing a desertification process and population migration towards urban areas. The *département* of Creuse in central France is a particular geographical case in point considered as being part of the French “empty zones”. As a result, during 1960s and 1970s, faced with the demographic explosion in Reunion and because the metropolitan rural countryside was being deserted, the government officially organised the emigration of some 75,000 Reunion islanders through the intermediary of the French bureau for the development of migrations in overseas territories.^1^

Two thousand and fifteen children were thus transferred to mainland France under the auspices of the French child protection institution and the French department of health and social affairs (DDASS). Social workers travelled up and down the island to meet families, whose situation had become vulnerable by social insecurity, inviting them to place their children first of all in care homes in Reunion. But these facilities were lacking on the island, and there was frequently pressure from the department of health and social affairs on the parents, who were often illiterate; this eventually led the parents to willingly allow their children to be sent to mainland France. These parents were given the impression that their children’s placement was only temporary and for their own good. However, the document they signed, without having been able to read it, was a seal of approval for their children’s definitive separation from them, which was made even more definitive when a decree dated October 15, 1960 (Debré) stipulated a ban on any claim: anyone claiming dysfunction was to be reprimanded, sent to prison, or excluded from French territory. From then on, these children became Wards of the French State (Ascaride et al., 2004). The communist party criticised this policy of forced migration, which also affected adults. They accused Michel Debré of “organising human trafficking” by sending Reunion workers towards mainland France (Eve, 2014, p.24).

This decision was kept silent for a long time (Cherki, 2006). However, on February 18, 2014, The French national assembly proposed a motion, calling for in-depth historical investigation of this affair, and its diffusion as they believed that the State had failed in its moral responsibility towards its wards. A commission of experts was created in February 2016 and a report with proposals for the provision of accompaniment for the former minors from Reunion was expected for 2018.^2^ This commission, composed of a sociologist, a historian and a geographer, was not asked to assess the psychological repercussions of this “transplantation”. There was no psychologist. Furthermore, the information gathered in this report, most of it from archives, did not explore subjective aspects, i.e., the experience of this forced exile among those who had lived through it.

## Aim of the Research and Methodology

Within the framework of an ongoing study assessing the psychological repercussions of this very specific life experience, 13 interviews were conducted with people who had been transferred to mainland France between the ages of 2 and 15.^3^ These interviews were made possible by the Federation for uprooted children, under the auspices of the DROM (overseas territories) and took place between 2016 and 2017. There were eight women and five men, presently aged between 47 and 67. The average age was 58 (Table 1). Seven interviews were conducted with people from Reunion living in Creuse, a French *département* they had never left since their arrival in the years 1965–70; three interviews were conducted in the Paris region and three others in the south of France, where these six people were residing. Among the 13 people, seven of them were aged 2–7years when they arrived in mainland France, three were 9–11years of age and three were 12–15. There was only one specific “family” situation; one woman, born in Reunion, arrived in mainland France when she was 2 with her mother who was then aged 17.^4^ The average age of the interviewees at the time of their separation from their parents was 6years. The average age at the time they left for France was 8years. Thus, the period of instability for their placement – involving many separations – lasted an average of 2years (Tables 2, 3). The 13 people interviewed experienced between two and seven separations, with an average of 3–4 separations.

**Table 1:** Participants’ dates of birth.

1950	1	1958	1
1952	1	1960	2
1953	1	1963	2
1954	2	1967	1
1957	1	1970	1

**Table 2:** Age at 1^st^ separation.

3months	1	7years old	1
2years old	2	9years old	2
3years old	1	10years old	2
4years old	3	13years old	1

**Table 3:** Age at the time of their exile to mainland France.

2years old	1	9years old	2
3years old	2	11years old	1
4years old	1	12years old	1
6years old	1	13years old	1
7years old	2	15years old	1

The research methodology was based on one already used in our previous study concerning a particular population’s life experiences and outcomes, those of Jewish children hidden in France during World War II (Feldman, 2009), who had lived through collective events that necessarily had an impact on each individual’s subjectivity. The development of a clinical analysis model led us to use it in other situations so as to gain the understanding of these situations in the context of contemporary psychopathology, as experienced by these Reunion children who had been exiled or “transplanted” to mainland France.

In a first step, a qualitative analysis of the narratives enabled the detection of what became of the traces of trauma at different ages, which proved particularly visible at key moments in life (Feldman, 2019): adolescence, maternity, parenthood, retirement, old age, becoming grand-parents… These ages can be seen as milestones that are often periods of psychological upheaval, rendering the subject vulnerable, or conversely, they can be levers for potential change. In the case of the Reunion children, the same periods: adolescence, maternity and parenthood were particularly difficult milestones.

Our main hypothesis was as follows: children exiled from Reunion Island to mainland France experienced a series of traumatic events that altered the process of their development, and there are traces after 40years. For the definition of trauma, we refer to a definition by Terr (1991) calling on Type-II trauma. This author distinguishes the effects of a single traumatic event, which she terms as Type I trauma, from the effects of a traumatic event that last over time and can be repeated (Type II). Our objective was to gather material that concerning the different problems specific to the unique situation of these children exiled to mainland France. What we have gathered are in fact life trajectories.

## Data Collection Method

The decision was made to conduct a single interview. The mean duration of the interviews was 2.30h. The shortest lasted 1.30h and the longest 4.30h. A single investigator (M. Feldman) conducted the interviews. The choice of conducting a single interview was motivated first because the study was an initial exploratory study that aiming to identify salient processes, and second in order to take account of the defense mechanisms coming into play. Indeed, at the time of this research, the expert report by the committee appointed in 2016 had not yet been issued to the Ministry for the overseas territories. It thus seemed to us more appropriate to wait for this first political step before envisaging interviews running over the long term. This ethical choice was intended to protect subjects from possible psychic decompensation in a period of ongoing uncertainty as to the political consequences of the issuing of this expert report to the French government.

Using semi-directive interviews, we asked each person to tell us about the events that they had been through. The questions were about family history and an attempt was made to go as far back as possible into the past.

We sought to identify the following: the experiences of children and parents on Reunion before their forced exile, the conditions of their separation, where they were placed, their experiences of nurseries and/or care homes and/or foster parents on the island before their departure, their journey from Reunion to mainland France, their arrival in mainland France, the conditions of their reception and placement, as well as their experiences of becoming an adult, taking account of key ages (adolescence, marriage, parenthood, becoming grand-parents and retirement), and any return to Reunion and possible reuniting with their biological families.

The interviews were conducted with the following chronology: life on Reunion, before exile: with the biological family and in foster/care homes; the journey to mainland France; life in mainland France: in care homes, with foster families and/or adoptive families; experiences at the different key age milestones; relationships with Reunion today; any reunion with their biological families; and difficulties encountered in the course of their lives.

After transcribing the interviews in full, two types of analysis were carried out: “biographical” and cross-sectional. The biographical analysis enabled and individualised narrative to be produced for each trajectory taking into consideration their fantasised reconstruction in time T of the encounter. In this analysis, we identified items that were common to all the narratives, reflecting the fact that everyone had been made vulnerable and had suffered consequences of the trauma.

The cross-sectional analysis compared each of the 13 observations item by item, according to a complementarist approach (Devereux, 1972). It also provided a psychoanalytical, historical and anthropological perspective. We conducted a fine analysis of the content of each interview, identifying themes and meta-themes that were common to the interviews overall, as provided for in the methodology of interpretative phenomenological analysis (Smith et al., 2009).

## Results

Across the 13 narratives, specific themes were identified in relation to traumas. These children were “exposed” to trauma many times before and after their exile to mainland France through their experiences as “transplanted” children. We were thus able to identify numerous vulnerability factors and the consequences of these traumas on their psychological development and their experiences of becoming adults.

### Vulnerability Factors

They have been termed as “exposed” children (Moro, 1989).

In the mythological sense, to expose a child means to put him/her in a hostile environment, so that he/she is confronted with danger, or even death. This concept implies the idea that being exposed to an extraordinary risk can either lead the child to become extremely vulnerable, or on the contrary, to acquire exceptional potential.

#### The History of the Island Rendered These Children’s Families Vulnerable

These children were vulnerable even before their “transplantation” because of the history of Reunion itself and that of their families.

Payet (2001, p.179) stated that the population of the island formed over 300-year period from a wide variety of ethnic groups. She explained that these people who were French citizens were from France, and also from Madagascar, Africa, India, China and the Comoros with their cultures, their beliefs and their languages, forming an “ethnic mosaic”. The population on Reunion was thus forced to adapt and integrate, and by doing so, lived through a number of bereavements and renunciations, not forgetting the issues of colonisation and slavery up to 1848. Every Reunion family therefore has a complex historical past and has been affected by it. Each family has had to deal with their own particular heritage, their cultural representations and their system of kinship, and, in particular, the naming of people (how the name is written, attributed, transmitted and what its function is; *Ibid*, p.180). Ghasarian (2002) characterised the complexity of Réunion society by the presence of three driving forces: acculturation, creolisation and cultural re-invention.

Beyond this painful collective history, these children were vulnerable because of the family situation they were in. Most children came from families with considerable social insecurity. Mothers were often single or had children from several relationships. In the interviews collected, there were often mentions of single-parent families, illness, violence and instability.

#### Filiation Breakdowns, Multiple Placements on Reunion Before Exile and III-Treatment

Filiation breakdowns correspond to the separation of children from their parents.

These children experienced a first separation, the separation from their parents, which is particularly painful. For children, separation is a source of anxiety, loss and abandonment, particularly, when the occurrence is sudden and abrupt. Furthermore, child–parent separations more broadly involve separation from familiar places and smells. Children’s ability to keep a representation of a loved one alive is limited when they cannot see or talk to this person. Winnicott (1984, p.68) tells how everything seems to be all right for a few days or a few weeks, until suddenly children do not know whether their mother really exists anymore.

From this first separation, followed a series of placements and frequent ill-treatment that these children experienced in childcare institutions on Reunion.

Jean-Paul, born in 1970, was exiled at the age of 3. He reported having been abducted at 3months old, being cared for by different nurses before a nursery fostered him until his journey to mainland France, where he was adopted. Born in 1967, Maryse was exiled when she was 4, after having been separated from her mother at the age of 2: “They came to tear us apart, yes, that’s exactly it, they came to tear us apart”. D. told me that she remembered the day when the social services came and remembered being traumatised because she was 10years old “and everything happened in the midst of crying and howls, when the social services came to tear us away from the family, who never saw us again and were never given any sign of life from us. From this foster family, we were taken into a nursery in Saint-Denis. We stayed there for 2years”.

Madeleine was born in 1957. Her mother died when she was 9months old, possibly following an accident and her father died after a “revenge attack”. She and her sister, 4years older than her, then went to live with their grandmother who died of TB. She was then placed in the Notre-Dame-des-Neiges orphanage and later placed in a religious institution. Before her departure for mainland France, she was placed with a foster mother who was “a child-abusing vixen,”… for 5 or 6months. She was exiled at the age of 9.

Born in 1954, Gérard said that his mother had died after his younger sister was born and his father had died when he was 11. He had been raised by his grandmother then placed in foster care on Reunion before leaving for mainland France at the age of 12. Before leaving, he spent a year in a home; he remembered: “they used to set the dogs on us if we did not keep in line. There was physical abuse and everything”.

Henri was born in 1953 of an unknown father. He remembered that his mother was often ill. When he was 5, Henri and his brother, who was 1year older, were “abducted” to be placed in a nursery in Saint-Denis for 1week. Henri was then sent to the *Plaine des Cafres* home and then to a foster mother. In 1965, his foster mother told him that is mother had just died, and the same year, the placement with his foster mother ended. He and his brother were taken into a home in Saint-Denis. He remembered that they had forced him to sleep with the pigs because he wet his bed. He said that he did not understand the staff’s attitude towards him and expressed his memories of the suffering he had endured in foster care on Reunion Island.

Violette was born in 1952. She remembered playing with her brother in the street when a woman driving a Citroën 2CV stopped to give them sweets. She made them get into her car. Violette was 12years old. Both children were taken to a foster care centre in Saint-Denis, where they were separated with no explanation. She said she was then sexually abused.

Born in 1950, Suzanne arrived in mainland France when she was 15years old, after having been placed in different facilities on Reunion from the age of 4: nursery, children’s home, religious institution and with a “child-abusing matron”. Over the course of her placement history on the island and in metropolitan France, Suzanne was placed in seven different locations.

Following an often brutal first separation from their families, other separations occurred and children were often placed in foster care or in nurseries on Reunion before they left for mainland France, where they experienced further separations until, for the youngest children, they were finally adopted.

These separations are the very roots of the subjects’ internal discontinuity, with a loss of the feeling of their permanent existence, vital for their development (Winnicott, 1987), because it forms the basis of structural stability that enabling children to build their internal world and acquire an identity (Houzel, 2016). Instability generates anxiety, which overwhelms the child. Therefore, any internal or external changes that children undergo lead them to perceive a distressing incoherence in their internal unity. Some children are subjected to chaotic, even disastrous experiences, and they have to grow up with them. These experiences can lead to the risk of depression, depersonalisation and attachment disorders in the long term. Furthermore, the age when separations occur is a particularly important aspect to be considered, since the impact of any breakdown depends on the developmental stage reached by the child. The younger is the child the greater is the child’s dependence on the contributions of external reality. The absence of a healthy primary object-relation, the absence of language and of the ability for symbolisation can potentially undermine the constitution of a secure attachment.

#### A Policy of Deception

Vulnerability was compounded by deception, which played an active role in this policy of “transplantation”: the promise made to the parents that their children would return and would be able to study in mainland France, a promise that was not kept. The children were also told lies: before leaving Reunion, they were shown beautiful pictures of France and they were promised “skiing holidays” and “Christmas celebrations”. When they arrived in mainland France, the French institutional representatives often told them that they were orphans or that they had been abandoned.

Marie-Claire, born in 1958, who was transferred to mainland France when she was 9 and half years old, remembered: “So, I found myself on that plane, completely lost, with children crying around me”.

Jean-Roger remembered being very scared in the plane and screaming. Marie-Claire continued: “You’re an orphan, you do not have your parents any more, you do not have a family any more, no brothers, no sisters: I was told I was an orphan… She told me: “you are an orphan with no father, no mother,” that is how I got to know it: “you are an orphan with no father, no mother, you do not have any parents, you do not have any brothers or sisters”.

Jean-Paul told how his adoptive parents always told him that he was from Reunion. The only information they were given was that his mother had abandoned him because she was very young and very poor.

#### Breakdown in Affiliation Links

Upon arrival in mainland France, separation from their island and their cultural system was materialised by the geographical distance and also by the ban on their Creole language.

Marie-Françoise, who was born in 1960 and taken to mainland France when she was 3, reported that she had been beaten for speaking Creole: “But I did not know I was speaking Creole, I actually had to be told by a woman from Reunion that I spoke Creole”.

When Jean-Roger arrived at the age of 7, his adoptive parents taught him how to forget Creole and speak French by “slapping [him] round the face”.

Jean-Paul was exiled when he was 3years old and said he had no memory of anything before 6years of age, which is when he started to speak. Between the ages of 3 and 6, he had become mute. He remembered having received numerous speech therapy sessions and having faced difficulties at school.

In addition, the Church took an active part in these separations from their origins. Indeed, these Reunion children were either *yabs*, *malbars*, *cafres*, *zarabs* or Chinese.^5^ When they arrived in France, they were christened, and thus converted to Catholicism.

Marie-Françoise, who was transferred to Creuse when she was 3years old and adopted 3years later, is now called as Claude: “there was [my name on] a plaque that was fixed to the wall above my bed, because in those days, there were plaques with saints on them, it was the way with saints. They were given for birthdays and we had to go to Sunday school”.

Marie-Claire said: “Mass every Sunday, Sunday school with pervert priests”. She added: “and there are people I have met up with again from St. Clar who have told me exactly the same things”.

#### Humiliation, III-Treatment and Sexual Abuse

Humiliation, ill-treatment and sexual abuse before and after their journey were for most of the respondents among the traumatic effects of exile. Besides ill-treatment endured in children’s homes and with foster mothers on Reunion, mainland institutions perpetuated the ill-treatment: for some boys, like Henri, they were used as farm boys and the eight women included in the study all endured abuse.

Upon arrival in the Guéret children’s home in Creuse, some described their reception as depersonalised, almost as de-subjectivised: “we were put in a room naked, with just our pants on, I could see a big steam machine and they disinfected us with it”. “There were so many children that they had to put mattresses in the corridors”.

When Valérie was 3years old, she was sent to mainland France with her siblings. She had no memory of her life on Reunion, she could only remember one thing: “I can see myself, I can see us on that plane, with a whole load of other children and I remember feeling completely lost”. When she got to the children’s home in Guéret, she remembered sleeping on a mattress on the floor in the corridor. Other fleeting memories came back to her and were linked to a hospital stay when she was treated for problems of undernourishment: “I can see myself hanging onto the nurses’ skirts”. When she came out of hospital, she was separated from her siblings and placed in a foster family for 4years, during which time she was subjected to abuse: “The only place I felt safe was under the Table. I used to spend a lot of time under the table. The father of the family used to beat me”. She was frightened. Occasionally “a white couple” used to come and visit her and would bring her dolls and presents. She remembered that the foster family’s daughter used to systematically throw her dolls and her toys down a well.

Marie-Françoise also experienced abuse in her foster family: “I’d say 3×2=8, but I really did not deserve to be slapped for that,” or again “when I grew older, I had friends, and when I saw how they were being treated, I thought: there’s a problem here, because when my friend does something wrong, she does not get a slap from her mum, she gets reprimanded, whereas when I answer 8 instead of 12 or break a glass, I find myself with… I get punished on top of that, which is all the better on dark skin because you cannot see the bruises”.

Henri arrived in Guéret in October 1966, after having transited through Paris, where “they were sorting all the kids”. After 3weeks at the Guéret children’s home, “on November 22, I was placed with well-to-do farmers on a farm…” where he was treated, he claimed, as “a slave”. He used to sleep on heaps of grain in the barn; he would wash with a basin and a bucket outside the house, “in summer as in winter”. “I was working 7days a week, from six in the morning to ten at night, with no rest or anything. Those people were paid to exploit us (…) I was beaten every day”. Despite his plea, he was not allowed to continue his schooling.

Marie-Claire, after having resisted adoption three times, stayed in the children’s home where she had been placed after her arrival: “I was employed for 10years. The first 2years, they used me as a cleaner. Then, I stayed 13years as a monitor. And, the following years I ended up replacing all the monitors who went on leave for specialised training. In fact I was pushed around as a stop-gap”.

Maryse arrived in her foster family in Brittany when she 4years old. The first memory she had was this: “I remember I used to wet my bed. To make me understand that I had to stop, she, my mother, put my knickers on my head and made me walk round the block of houses. Well, I did stop wetting my bed after that”. (…) Reminiscing about her foster mother: “I remember a very violent woman, whether in actions or words, she used to say things like “be grateful we pulled you out of that shit” (…) In my mother’s eyes, I was worthless, and whatever happened, I would end up on the street”.

Madeleine recounted her “ordeal” when she arrived with her sister at a childless adoptive couple’s home: “The ordeal started because this man, every night at 3 o’clock in the morning… it was a house where there was a wooden staircase, so we could hear the stairs creak. We used to sleep in the dining room because we did not have a bedroom. We only had a single bed so we used to sleep next to each other. So every night, he would come down and interfere with my sister and would end up slapping her. He would use words, of course… of a sexual nature. As for me, I was afraid, I could not breathe. From then on, I thought to myself: You have to play dead so that he cannot hear you. I started being unable to sleep, the same for my sister. It felt as if I was being raped myself as I was just next to her”.

Questions about their origins were always present and re-activated by the mockery and discrimination endured, particularly because of these children’s skin colour, different from the other children: “They used to call me chocolate, little *darkie*” one person reported. “A bus from the village used to pick us up in front of St. Clar, and we were considered as “social cases,” mental cases (…). I could not stand being insulted like that (…) In 1969, when you came to a small village, there were no coloured people”.

At school, near Guéret, as Valérie was the only child with dark skin, she endured discrimination. They used to say to her “Snow-White, you dirty *nigger*, did you wash this morning?” In secondary school, one pupil wrote a song about her: “the little *nigger*’s peed in the water…”. Discriminations were such that she refused to eat at the canteen for 4years.

#### Chaotic Adolescence

During adolescence, life becomes particularly difficult. It is a period of psychological upheaval; it is also a time during which ontological issues arise: Whom do I resemble? What do I belong to? Who do I want to become? (Mansouri, 2013). There are questions about the meaning of belonging. This period can be very painful. It is a time when the Oedipus complex is reactivated and when adolescent subjects are supposed to de-idealise parental images introjected during childhood, and at the same time, they are faced with the issues of the family heritage (*ibid*, 2013). For these children, for the younger ones in particular, this can correspond to a dislocation of introjected parental images. They thus grow up with these rifts, these discontinuities, these gaps that they have to fill almost impulsively to acquire a semblance of security, which is essential to survive and which necessarily comes with pathological defences.

Marie-Claire: “Whenever I had my period, I did not know what was wrong with me, why was I bleeding?” (…) “Every Christmas, we would be alone in bars. Where was everyone? Well, they were all at home with their families of course, celebrating. And we felt lost. At that moment, I realised that something was wrong… in my life, because I had an abortion, in fact I had more than that”.

Marie-Françoise remembered that adolescent period: “I was in secondary school. Oddly enough, I was placed because I was one of the turbulent children”… “I used to do sports, athletics, competitions. They stopped me doing all this”. That period also corresponded to the divorce of her adoptive parents. “I was 16 and I fell in love… he became my stepfather”.

During that time, Jean-Roger experienced many years of drifting and instability both geographical and professional…

#### The Complexity of Reaching Parenthood

These difficulties in becoming a parent are linked to the separations, to a fluctuation or an absence of a parental model, to the instability of their relationship with the primary object, and these difficulties are reactivated by their new parental status. The eight women in the study all experienced domestic violence and separated from their partners.

Maryse raised her child with a violent man for 4years. She then met another man who left when she was pregnant, and she then had two children with a third man whom she married twice, as she divorced from him once during that time.

“Violence is perpetuated,” admitted Marie-Françoise. For Maryse and Marie-Claire, domestic violence followed on from their adoptive mother’s violence or from their adoptive father’s sexual abuse. For one, her partner was violent. For the other woman, violence was rife towards her children, who were later placed in childcare.

Violette was married to the first man she met when she was 22years old. Her husband was an alcoholic and a violent man. She managed to get a divorce after she had had three children with him. These children have their own children today. The relationships with each one of them are very difficult, almost non-existent. She does not understand. Suzanne acknowledged the fact that her history made parenting difficult: “I did not know how to love my child… My daughter said: “mum, you never gave me any cuddles,” I said: “it would have been easier if I had had cuddles myself”. I said: “I never had a mum who gave me cuddles, it would have been easier had I received maternal love to be able to give it to my children”. I did not know. This is why it’s so hard. I was never brought up in that way, I was brought up in a violent environment”.

Henri was sad to relate the difficulties he had with his sons: “With my elder boy, it’s not great. We cannot talk that much because there’s a problem of alcohol. He drinks a lot. With my second son, it’s the same, he drinks, he takes drugs and he is violent, so I do not see him any more either. I keep saying: “I brought children into the world and this is where we are now”.

#### Painful Homecoming With Reunion

Many years later, forging ties again with Reunion became necessary, but this homecoming reopened painful wounds on either side.

Marie-Claire discovered her own history when she was 30years old: “I could see that I had a mother, that I was not an orphan with no father and mother. Because my mother had signed… this document so that I could come to France… to study. Which means that St. Clar knew I wasn’t an orphan. I could become an orphan in a flash, because my mother signed the document. But my father did not want me to go. There was a thumbprint… I mean, he had written: I do not want my daughter to leave. So why was there a thumbprint? It was at the Saint-Denis prefecture. My cousin had arranged for me to get in touch with my mother. Feeling guilty, it took my mother a year and a half to respond. She was ashamed. So it was I who decided to meet her in 1987 (…). It was difficult for me to see my mother. In fact, I did not want to. I thought: “what am I going to say to her? It’s going to be really, really hard (…). She told me what had happened when I went to see her in 1987. She said: “you know, I found you. The Social Services came to see me, they made me sign a paper so that you would go to *Plaine des Cafres*, so that it would give me time to get better and for you to get an education, and I would be able to come and get you a year later”. She continued her narrative, stating that her mother had wanted to get her daughter back after 1year but that she was no longer there.

When Valérie was about 30years old, she learned of one of her brother’s suicide; he was 32years old. It was like a “trigger” for her, as it prompted her to go back to Reunion. Her adoptive mother was not very keen, but in 1992, she decided to go with her brother and one of her sisters. When she arrived on the island, she said she was in “total denial,” she felt guilty and tormented by a conflict of loyalty towards her adoptive parents. Her first reaction was: “When I see the poverty in the family that claims to be my family, I say to myself “how lucky I’ve been to live in mainland France”. She returned to mainland France and claimed to be a pure native from Creuse. Valérie then lived through a period of great instability. She said she moved house around 15 times. When she was 43years old, Valérie returned to Reunion on paid leave, as usually granted to French overseas civil servants working in mainland France. When she returned, she asked her civil service department to be transferred to Reunion, which was granted. So she lived on the island for 9years, particularly devoting her time to in-depth research on her family roots. But on Reunion, her history was seen as taboo. Her family refused to speak. A friend of her biological parents refused to talk to her. One of her cousins also kept silent, claiming: “the truth should be spoken once people are dead”. One day, she met a *gramoune* ^6^, who started to cry upon seeing her: “you look so much like your mother!” But she refused to tell her anything. With a lot of determination, Valérie started to uncover fragments of histories, particularly about her mother who had apparently committed suicide at 28 because she had yet again become pregnant. Today, Valérie continues to search for information, but tongues remain tied. She has now returned to mainland France and has been retired for 2years. She is involved in movements for the recognition of suffering among the ex-minors transferred from Reunion to mainland France, and in 2017 she was still hoping to go back to Reunion to continue her search. Her hypothesis was that she probably was a stolen baby. Her identity was erased: “on the civil act, I was born in Creuse, I have two names, two first names, two baptisms, two birth places”. In her file, there was no document proving any abandonment.

Maryse went to Reunion for the first time in 1988, where she met her uncles, aunts, brothers and sisters. It was only in 2014 that she realised she had been adopted during the time Michel Debré was implementing his policy, and that she was not the only child involved. The family had never told her anything. She collapsed completely. Today, she continues to learn more and more about her childhood past.

Jean-Paul stayed on Reunion on a number of occasions; however, it was only in 2017 that he learned that he had been abducted from his mother when he was 3months old, because he was shown a document, which had been drawn up by the social services in which maternal abandonment was mentioned, but without any trace of her signature.

The experience is therefore painful and the psychological repercussions are great at individual and inter-subjective levels. While most of the children did not set roots in mainland France, they did not grow roots in Reunion either, 40years on. Quite the reverse, the early experiences of abandonment seemed to be reactivated.

### Consequences of Trauma

#### Specific Symptoms Among Children Exiled From Reunion

The people who were interviewed all shared a deep feeling of abandonment, throughout the many separations: from their parents, from the care home/nursery, from the foster home in mainland France, from their adoptive families, etc. A pathology of bonds is at the heart of their suffering. These “exposed” children became children who were “major relational burn victims” (Lamour and Barraco de Pinto, 2013, p.119).^7^

Their symptomatology covered a large spectrum: affective and/or professional instability, emotional lability, nightmares, fear of the dark, fear of being in a closed room, hyperactivity, addictive disorders and repeated suicide attempts, which, for most of them, started during adolescence and were often followed by psychiatric care with feelings of never knowing, where they were supposed to be: “I was never where I should be,” “where should I be?” in the words of Marie-Françoise. There were feelings of anxiety, feelings of being nowhere, depressive episodes, somatic disorders such as ulcerative colitis and psoriasis. “I’ve always had the feeling of being alone,” said Maryse.

Today, Valérie continues to have nightmares: she is being “chased,” she has to “hide,” she falls “into a hole,” “no matter how much she tries to hide,” she “continues to be chased”. She is always afraid of closed rooms. She said that she had attempted suicide on two occasions. Valérie was followed by a psychiatrist and had treatment. She went through periods of depression. Because of the abuse she endured and because of serious health problems, she was considered as a disabled worker. Suzanne fights every day to live: “I take pills to sleep, I take pills to get up, I take pills to get through the day” (…). When talking about the children’s experience of their exile from Reunion to mainland France she stated: “Even if we are alive, we have been killed in our souls”.

For some years, after every return to mainland France from a stay on the island of Reunion, every time they return from a general assembly of the ex-minors from Reunion, many of them experienced a state of acute vulnerability: depression, suicide attempts, instability. Great vulnerability is experienced on the occasion of every process of separation, requiring psychological readjustment: adolescence, maternity, separation from a partner, their own child’s adolescence, return to Reunion, return to mainland France…

Marie-Françoise remembered: “I stayed in Reunion for 5months. I came back to France… I had a breakdown… I was hospitalised in a psychiatric ward (…) Every time I come back from Reunion, every time I feel lost. They give me treatment, and every time they are antidepressants, Xanax, Stillnox and so on”.

Marie-Claire had ulcerative colitis when she was 20years old, at the time she was leaving St. Clar. She had regular bouts of depression, especially after she had read through her file that had been sent from Saint-Denis, and after a film had been made of her story, in which she had taken part. In August 2015, she said she had been prescribed antidepressants, which she had taken for 4days, after which time she had stopped them because she felt like “a zombie”.

It is well-known that filiation takes shape *via* bonds and continuity and also by separations and discontinuities (Konicheckis, 2001). The quality of the fantastical and psychic development can make this discontinuity bearable and acceptable, or on the contrary, it can be traumatic and contribute to loosening the bonds. However, when a psychological breakdown occurs, as is the case with these children, and when their development and bonding process is hampered, discontinuity creates havoc. Every separation is thus experienced as an aftermath, which can sometimes lead to psychiatric decompensation.

Being removed from the cultural (Feldman, 2016), familial and geographical envelope, to which is often added physical and psychological violence, raises the issue of the legitimacy of existence, which is never resolved.

These observations are also reported among the formerly displaced children for reasons relating to public policies: Inuit children having undergone acculturation and forcibly placed in Christian children’s homes (Mishara and Tousignant, 2004), half-cast children from Ruanda-Urundi removed to Belgium (Heynssens, 2012; Hennes, 2014), children stolen under Franco’s regime (Vinyes et al., 2012) or again thousands of young Eurasian girls removed from Indochina to France between 1940 and 1970 for purposes of cultural assimilation *via* FOEFI (Fédération de l’Oeuvre de l’Enfance Française en Indochine). The mothers were obliged to sign a “*certificat de décharge*”, which drastically reduced their rights towards the children, and siblings were systematically separated (Denéchère, 2020). These trauma and the post-colonial policies faced by these children had devastating effects on their subjective construction.

#### Intra- and Inter-Generational Family Disorders

When there are reunions with biological parents, they can be particularly difficult, even painful. After 40years of silence, absence and lies, reunions are extremely difficult, sometimes impossible, with a persistence of the unsaid. From the point of view of these children who have become adults, how could they face finding their parents or other members of their families after believing they were orphans all these years? And, for some, the belief that they had been abandoned by their parents.

As for the parents, these reunions lead to a reactivation of past pain. If they are still alive, they have grown old and adapted their lives to this absence. The mourning for the loss of their motherhood (Racamier et al., 1961) and their parenthood is therefore destabilised, or even undermined. Wounds from the past are reopened. The return of their lost and often forgotten children is therefore painful to accept. These children have become strangers in the eyes of their biological parents and are often perceived as revenants. It is a real shockwave on either side of the family, which is made even greater because these reunions are unprepared. Even if some ex-Reunion children took the initiative to return to the island, often with considerable apprehension, their parents on Reunion did not share the same time-line. For some, the surprise of this return is a bolt out of the blue.

Some children wish to settle down on Reunion. But their return triggers an aftermath effect on the history of the Reunion people. How can they integrate these returning migrants? How can these migrants adapt? This is all more true because they have been de-named, renamed and have been subjected to an acculturation.

Relationships with adoptive parents often remain difficult, especially when they played a part in the separation, chose to remain silent and ultimately did not do the right thing.

Difficulties with these children’s own children are of prime importance. Their history is not elaborated. Past experience is manifested in the expression of non-metabolised, raw objects, and their children have become empty vessels of the parental history rather than inheritors (Feldman et al., 2016), and this is all the more true because the State has never acknowledged these events. Therefore, the encystment of the parental past is handed down to the descendants without any processing. Among the descendants, a certain form of drift or instability in affiliation can be identified for some, for whom the parents tried to be parents despite everything, while for others dysfunctional parenthood was such that they were taken into childcare. History repeats itself.

Regarding her daughter, Marie-Françoise said “I’m hoping for an event that will make us grow closer to each other”. A crisis of transmission is experienced by both generations.

Only Jean-Paul, adopted by a kind and loving couple, appeared not to have experienced these difficulties with his children, he even involved them in the search for his filiation.

## Discussion

### After Enduring an Accumulation of Traumas, These Children Became Adults Deprived of Their Inheritance and Whose Identity Had Ceased to Exist

The events endured since leaving Reunion and up to the present day have produced an accumulation of traumas, defined by Khan as the accumulation of repeated failures of the maternal object in its role as “a protective barrier of the auxiliary self” (Khan, 1976, p.74). They have an impact on the children’s development and on their becoming adults (Feldman, 2013).

While the interviews collected are not representative of all the “Creuse children,” the adults encountered seemed bound to reproduce the painful sequences of their childhood experiences and exposure to an accumulation of trauma. The Freudian concept of compulsion to repeat (1920) describes the way in which the traumatised subject, in an aftermath effect that is almost a compulsion, attempts to gain control over the disorganising effects of the trauma, and paradoxically to re-experience them as a result of an attraction towards the intensity of perceptions specific to the initial traumatic episodes (Freud, 1920/2001).

Beyond the notion of disaffiliation induced by childcare placement, it is the very notion of filiation that is being damaged, operating *via* a process of de-subjectivation leading to a suspension of identity. This damaged filiation is the result of the effects of de-filiation and disaffiliation and of a historical policy of de-subjectivation.

#### The Effects of De-filiation and Disaffiliation

These children endured de-filiation from their biological family and disaffiliation by separation from their island and their Creole cultural system. For children’s secure psychic development, issues of filiation and affiliation are central. Indeed, a child’s birth occurs within a particular genealogy, history and geography. As a child of a father and a mother, the child necessarily relates to their filiation: paternal, maternal parentage, family, and groups of people beyond to whom they belong; hence, the importance of filiation, which is a pre-existing base for any human being even before birth. The notion of filiation is used here in the way that Michael Housemman defined it, as quoted by Jean Guyotat (1995), in an anthropological perspective. According to him, filiation is governed by a genealogical principle whereby there is a notion of belonging to a certain community, comprising people who are said to have a common ancestor (p.8). Jean Guyotat (*ibid*) distinguished three aspects that were inherent in these filiation ties. The first aspect relates to established, official filiation, with the transmission of a name in the setting of relations with others, as defined by social forms. This mode of filiation takes discontinuity into account in the order of generations. Guyotat then describes a metaphorical and metonymical filiation. Between the body of the father, who gives his name, and the body of the child who receives it, a transfer of meaning occurs, a metaphor, whereas the body of the mother, which contains the body of the child, relates to close body-contact metonymic filiation, a blood bond or a bond of continuity. Finally, narcissistic filiation concerns the process of filiation. This bond is found within the notion of the filiation group, which supposes a reiteration of this bond from generation to generation, starting from prestigious ancestors (p.38). Given that each subject is assigned a place and a mission in the group they belong to (family and social group), filiation subjects them to the generational chain.

The term affiliation is borrowed from anthropology: it describes a belonging to a group (Levi-Strauss, 1973, p.132), whether it is cultural, social, ethnic, religious, etc. Filiation and affiliation together weave the individual into the continuity of generations.

Filiation is also defined by the name, which gives a legitimacy of existence since it has an origin, a function and a meaning. A name provides a sense of continuity in the existence of an individual who is inevitably caught up in the experiences of discontinuity and separation. It is a link inside the psyche, which defines the narcissistic function of paternity, whereby the continuity of the species and of individuals is preserved. Therefore, to change that name is to intentionally change the child’s destiny.

Finally, affiliation links subjects to social issues in the terms of a narcissistic contract, in the sense that this contract has to be narcissistically invested by contracting parties, subject and group (Castoriadis-Aulagnier, 1975). In the absence of a narcissistic investment, the child is in limbo. The violence he is subjected to is to be found at the point, where he is forced to renounce everything that constituted his former identity.

Exposure to these many geographical filiation and affiliation breakdowns proved destructive for the children from Reunion, and jeopardised their genealogical foundations (Ayoun and Tabone, 1994, p.28).

These children were thus subjected to a metamorphosis of their identities, which was difficult or impossible to accept. Furthermore, this disaffiliation led to a primary narcissistic fault, which sooner or later freezes the subject in his psychic functioning or draws him towards a collapse of identity (Allouch, 2001, p.38) that analytic therapy may not be able to defuse.

This breakdown was also accentuated by the lies told to both parents and children. Lies are the most devastating for children because they grow up believing they are protected by adults. As a relational mode, lies lead children to lose trust in adults, and to a loss of “life theories” (Bailly, 2007) – these “theories” (p.241) that refer to a sub-group of the symbolic field. That is to say, during the first years of life, children work on the elaboration of their personal development and form hypotheses on their origins, on gender differences, on kinship, parental roles, etc. Traumas will then occur and relentlessly overturn the theories that children build throughout their development. Their historical perspective is thus called into question.

Damage to filiation can also be found here in the zeal of social workers and decision-makers in childcare social services in working to implement a politically-driven operation. According to Jablonka (2006, p.227), child protection systems were in this instance hijacked by the State to impose a new culture on these children.

Finally, Ascaride et al. (2004) reported on the attitude of the Guéret foster home director, who at the time, seeing the children’s distress, wanted to take them back to Reunion during the holidays. He told how he had written a letter to Michel Debré, who had refused categorically. He himself was from Reunion and had introduced Creole culture in the care home: Creole meals, Sega music… This director was later dismissed.

#### The Effects of a Lasting Policy of De-subjectivation

This damage to filiation echoes that endured by their ancestors. The intention was to protect these children and to serve a political project, but they were subjected to damage to their filiation (Feldman, 2018), where it is easy to perceive the fallout from their colonised ascendants’ history, people who were slaves over a 200-year period: these people had to obey their masters, had to bear the name of their owners (Payet, 2001) and had to be practicing Catholics, the only authorised religion (an article of the *Code Noir* or “black code”). The children from Reunion who were transferred to mainland France, disaffiliated, adopted, also had to bear the name of their “new parents,” be baptised and go to catechism in a specific context. Many were exposed to violence. The inheritance of the alienating and denied violence in their collective history plays a role in their present-day individual histories. This could be linked to what Malika Mansouri (2013, p.165) recalled for Fanon, when in 1952 he took up a stance against the Algiers culturalist school saying that the alienation of the colonised people contributed to the deleterious individual psychological consequences of a situation of domination (Fanon, 1952). Traumatic markers of historical, familial and institutional violence were found among all the children, as was the impact on subjectivity.

#### Healing Aftermath Traumas *Via* Group Work

Issues of transmission, secrets, separations, conflicts, or mixed race brought on by colonisation and slavery are at the heart of the history of the Reunion people. The “transplantation” of these children and adolescents thus operates as an aftermath effect of this particularly chaotic history. Stories of multiple traumas experienced over many generations were heard from every subject interviewed. Because they are not transformed by the psyche, these raw traumas become potent as “subconscious residues” that accumulate and run through generations. They are non-elaborated raw elements that are brought to light by blanks, gaps or events that appear incomprehensible, or nonsensical, in the light of the subject’s history. By way of a “narcissistic contract” (Aulagnier, 1975), this process prevents descendants from cutting ties with their ancestors and enables a transformation of the potential encystments of the past. This concerns both the parents, who lived in poverty and who were robbed of their parenthood, and the grand-parents, or even the great grand-parents, whose suffering caused by colonisation and slavery has never been recognised by France.

Our method consisted in elaborating a reflection from the histories of the people we met, to be as close as possible to them, without trying to interpret their words from a set perspective. We tried to show that the idea was not to label them the way clinicians may do from a traditional nosographic perspective. Nor was it to say that they had a neurotic or psychotic structure. They are not psychiatric cases, which would make them pathological cases, according to a generalizing nosography. Diagnoses tend to freeze people, lock them in, and they do not allow them to evolve. Through an analysis of their very particular life trajectories, we have tried to show the impact of historical events on their development processes and how they have suffered specific traumas. The people we met were affected by post-traumatic symptoms. We took an interest in their individual histories, because which are part of their collective history, by taking into account historical, political and sociological aspects.

To be able to care for of these former Reunion children, clinicians therefore need to consider each history as part of a trans-generational, historical and familial collective narrative (Feldman, 2009; Mansouri, 2013; Feldman & al. 2016; Feldman and Mansouri, 2018). The group setting, i.e., talking or focus group, seems important to implement. Indeed, it enables the collective part to emerge from each participant’s intimacy. The idea would be to devise a multi-focal group led by two psychologists, to enable the diffraction of transfer and to enable clinicians to benefit from support when faced with the massive nature of the trauma experienced, and of the inherited trauma.

This group could integrate different disciplines (psychology, anthropology, history and sociology) and could use its group format for its containing functions, a focus of psychology for many years, especially in the setting of trauma. The group favours the restoration of subjectivity and elaboration, in particular because collective suffering compromises affiliations. Thus a plural presence can put things into historical perspective, leading on to a process of shared remembrance (Kaës, 2012, p.247). In addition, it can facilitate care that is better adapted to the cultural representations of the individual, envisaged in constant interaction with his or her birth affiliation.

Furthermore, with a transcultural approach (Berry, 1990) it will be possible to identify the processes involved in continuities and changes in play in the acculturation processes undergone.

In order for the collective to emerge in its relation with the intimate, everyone involved, including the psychologists, should introduce themselves with their affiliations, linked to their own collective past, so that a certain formerly repressed subjectivity can emerge within this articulation between the collective and the individual. The analysis of cultural counter-transference (Devereux, 1967; Rouchon et al., 2009) on the part of psychologists will help understand the elements emerging from the group.

Finally, a therapeutic approach could consist in the de-construction of numerous systems: the colonial system, the slave system, and also institutional systems, particularly child protection facilities, which operated for a long time on strong assumptions such as the supposed benefits of parent–child separations, the secrecy around origins, indenture at 13years of age and the development of a dependence bond between individuals and institutions (Feldman and Hazan, 2017).

## Conclusion

This initial study presents imitations, mainly for the lack of depth of the investigation, requiring further investigation in the future. Indeed, a single interview is not enough to identify the psychic processes at play. This exploratory research has nevertheless enabled the particular itineraries of a complex, collective history of assimilation to be identified. This entailed eradication from the cultural, linguistic and historical setting before arrival in mainland France, leading to devastating effects on subjectivity and the family dynamic.

These 2,015 Reunion children, and their own children, with their unique destiny call for clinical reflection to integrate all the elements of the current complexity mingling with the complexity of the past. Therefore, it is today clinically urgent to work towards the implementation of this group narration process, particularly as these narratives do not only concern those who lived through them but also their descendants. When raw elements are not transformed, the risk of their unprocessed transmission is significant, because the historical trauma persists, relentlessly trying to leave a trace. This trauma is organised in encrypted mode (Abraham and Torok, 1978), which alienates all freedom. The next generation therefore carries a “ghost” that haunts their memory and relentlessly undermines the familial psyche.

## Data Availability Statement

The original contributions presented in the study are included in the article/supplementary material, and further inquiries can be directed to the corresponding author.

## Ethics Statement

The studies involving human participants were reviewed and approved by the Ethics Committee of the UFR SPSE (Psychological Science and Education Science Formation and Research Unit) of University Paris Nanterre. The participants provided written informed consent to participate in this study.

## Author Contributions

MF is a researcher. MM helped the methodology. All authors contributed to the article and approved the submitted version.

## Conflict of Interest

The authors declare that the research was conducted in the absence of any commercial or financial relationships that could be construed as a potential conflict of interest.

## Publisher’s Note

All claims expressed in this article are solely those of the authors and do not necessarily represent those of their affiliated organizations, or those of the publisher, the editors and the reviewers. Any product that may be evaluated in this article, or claim that may be made by its manufacturer, is not guaranteed or endorsed by the publisher.

## Footnotes

^1^BUMIDOM (Bureau pour le Développement des Migrations dans les Départements d’Outre-Mer).

^2^I (first author) was auditioned on June 17, 2016 at the overseas Ministry during this commission to shed light on the potential psychological repercussions of such experiences. An expert report dated April 10 of that year evidenced the fact that this transplantation had taken place in compliance with practices and regulations operating in child protection at that time. Therefore, no evidence of child theft or abduction was found in the files.

^3^The interviews were carried out by the first author of the article.

^4^The subjects interviewed were signed the informed consent before taking part in the research.

^5^*Yabs* are descendants from Bretons, *Malbars* are Hindus or Tamils of Indian origin, *Cafres* are black of African origin, descended from slaves and Zarabs are Muslims of Indian or Pakistani origin. The French from mainland France are called as Zoreilles.

^6^Elderly person in Creole.

^7^A phrase taken from Lamour and Barraco de Pinto (2013).
